# An Evolution-Based Model of Causation for Aging-Related Diseases and Intrinsic Mortality: Explanatory Properties and Implications for Healthy Aging

**DOI:** 10.3389/fpubh.2022.774668

**Published:** 2022-02-18

**Authors:** Gilberto Levy, Bruce Levin

**Affiliations:** ^1^Independent Researcher, Rio de Janeiro, Brazil; ^2^Department of Biostatistics, Mailman School of Public Health, Columbia University, New York, NY, United States

**Keywords:** aging-related diseases, complex etiology, sufficient and component causes, Gompertz distribution, Weibull distribution, healthy aging, health promotion, postponed aging

## Abstract

Aging-related diseases are the most prevalent diseases in advanced countries nowadays, accounting for a substantial proportion of mortality. We describe the explanatory properties of an evolution-based model of causation (EBMC) applicable to aging-related diseases and intrinsic mortality. The EBMC takes the sufficient and component causes model of causation as a starting point and develops it using evolutionary and statistical theories. Genetic component causes are classified as “early-onset” or “late-onset” and environmental component causes as “evolutionarily conserved” or “evolutionarily recent.” Genetic and environmental component causes are considered to occur as random events following time-to-event distributions, and sufficient causes are classified according to whether or not their time-to-event distributions are “molded” by the declining force of natural selection with increasing age. We obtain for each of these two groups different time-to-event distributions for disease incidence or intrinsic mortality asymptotically (i.e., for a large number of sufficient causes). The EBMC provides explanations for observations about aging-related diseases concerning the penetrance of genetic risk variants, the age of onset of monogenic vs. sporadic forms, the meaning of “age as a risk factor,” the relation between frequency and age of onset, and the emergence of diseases associated with the modern Western lifestyle. The EBMC also provides an explanation of the Gompertz mortality model at the fundamental level of genetic causes and involving evolutionary biology. Implications for healthy aging are examined under the scenarios of health promotion and postponed aging. Most importantly from a public health standpoint, the EBMC implies that primary prevention through changes in lifestyle and reduction of environmental exposures is paramount in promoting healthy aging.

## Introduction

The most prevalent diseases in advanced countries nowadays, accounting for a substantial proportion of mortality, are aging-related diseases such as ischemic heart disease, stroke, several types of cancers, and neurodegenerative diseases including Alzheimer's and Parkinson's diseases. Aging-related diseases are characterized by complex etiology (i.e., involving multiple genetic and environmental causal factors) and increasing age-specific incidence rates with increasing age (1). Commonly used in epidemiology, the sufficient and component causes (SCC) model of causation is particularly useful for complex diseases, because it provides a convenient way of conceptualizing biological interactions (i.e., gene-gene, environment-environment, and gene-environment interactions) (2, 3). In the SCC model, each component cause (a genetic or environmental factor) is part of one or more sufficient causes, while each sufficient cause includes one or more component causes. A sufficient cause constitutes a minimal set of conditions that produce disease, and a component cause corresponds to “an insufficient but necessary part of a condition which is itself unnecessary but sufficient for the result” (denoted by INUS) (2).

In this article, we describe the explanatory properties of a model of causation that takes the SCC model as a starting point and develops it using evolutionary and statistical theories; we call it the evolution-based model of causation (EBMC) (1). The EBMC is applicable to aging-related diseases and intrinsic mortality (i.e., excluding deaths due to extrinsic causes such as accidents and infections) and explains several aspects of aging-related diseases as well as the Gompertz mortality model with its characteristic exponential increase in age-specific mortality rates with increasing age. After briefly presenting the EBMC, we describe in turn its explanatory power for aging-related diseases and its explanation of the Gompertz mortality model. In a separate section we consider the limitations of the EBMC and, finally, discuss its implications for promoting healthy aging by examining the scenarios of health promotion and postponed aging.

## The Evolution-Based Model of Causation

Starting from the basic characteristics of the SCC model, the EBMC is developed using the evolutionary theory of aging and the statistical theory of extreme values. Their fundamental concepts and arguments most relevant to the EBMC are summarized in Table 1. The EBMC involves two major developments. First, component causes are classified based on evolutionary reasoning. Genetic factors are classified in terms of the timing of their effects as “early-onset” or “late-onset.” Early-onset genetic effects (EOGE) and late-onset genetic effects (LOGE) are defined according to whether the expression of their causal role occurs, respectively, before or after the earliest age of reproduction in the population (i.e., the age at which the force of natural selection starts to decline, see Table 1). For our purposes, the relevant earliest age of reproduction in the population is that which predominated among human ancestors during a continued period of time over evolutionary history, which can be taken to be about 10 years (25). In turn, environmental factors are classified as “evolutionarily conserved” or “evolutionarily recent.” Evolutionarily conserved environmental factors (ECEF) are defined as having been present during enough time in evolutionary history for adaptation to their effect to take place. They are what we consider “part of nature,” implying that members of the population are exposed to these environmental factors early in life, such that it can be reasonably assumed that they express themselves before the earliest age of reproduction in the population. Evolutionarily recent environmental factors (EREF) are recent enough on an evolutionary scale so that adaptation to their effects has not occurred. They have appeared mostly over the last 200 years since the Industrial Revolution, the relevant implication being that environmental factors brought about by industrialization (pollution, chemicals, toxins) and the modern lifestyle (cigarette smoking, changes in diet, sedentarism) fall under this category.

**Table 1:** Theoretical background of the evolution-based model of causation; summary of fundamental concepts and arguments of the evolutionary theory of aging and the statistical theory of extreme values.

**The evolutionary theory of aging (4–8)**
**1. Among ultimate theories of aging, which seek to answer why aging occurs as opposed to theories addressing the proximate mechanisms of aging, the evolutionary theory of aging had its basic argument initially articulated by Medawar (6, 7). At first sight, aging seems paradoxical from an evolutionary perspective, because natural selection acting on individuals supposedly causes the evolution of increased, not decreased, fitness. Medawar's reasoning reconciled evolution with the fact that aging is non-adaptive**.
**2. In Medawar's thought experiment, if one starts by considering a theoretical potentially immortal and ever-reproducing population, one can envision that the older the members of this population are, the fewer there will be of them simply because they are exposed for a longer time to the hazard of death due to extrinsic causes such as accident, predation, starvation, and infectious disease, which prevailed throughout human evolutionary history. Thus, older individuals make progressively less contribution in terms of reproduction to the next generation, implying that “the force of natural selection” weakens with increasing age**.
**3. The force of natural selection is a measure of the intensity of selection on genes. If we now consider a population with a window of reproductive ages, a lethal mutation whose effect occurs before the earliest age of reproduction in the population is not passed to the next generation (meaning that the force of natural selection is maximum), while a lethal mutation whose effect occurs after the end of reproduction in the population would freely pass to the next generation (the force of natural selection is zero)**.
**4. Between those two ages, the force of natural selection declines with increasing age because of a decreasing contribution to reproductive output. In humans, due to the extreme dependence of human offspring during infancy and early childhood, the force of natural selection is dependent not only on reproductive output but also on transfers of food and care (e.g., parental care and help from others such as older siblings or grandparents) (5)**.
**5. From the preceding points, the central idea of the evolutionary theory of aging can be stated as follows: the force of natural selection acting on genes whose effects occur at a given age (i.e., age-specific genetic effects) declines with increasing adult age, such that aging results from the accumulation of deleterious mutations with late age-specific effects**.
**6. This evolutionary process accounts for the progressive deterioration of physiological function characteristic of aging (9, 10). Aging-related diseases result from the same general process, but without necessarily sharing proximate mechanisms with aging (11–13), as initially put forward by Medawar (7) with respect to cardiovascular diseases and cancer**.
**7. Starting with Fisher (14), and mostly through the works of Hamilton (4) and Charlesworth (15), population genetics has provided the evolutionary theory of aging with a mathematically explicit basis. Hamilton (4) formally showed that the force of natural selection acting on a mutation that reduces survival decreases with age starting at the earliest age of reproduction in the population. This was illustrated using data of the population of the United States (16)**.
**8. Two population-genetic mechanisms have been proposed for the evolutionary outcome of aging. Mutation accumulation is the passive accumulation of mutations with late-onset deleterious effects (when selection is weak or absent) (7). Antagonistic pleiotropy is the active fixation of mutations with early beneficial effects (when selection is intense) and late deleterious effects (17). Experimental and comparative biology studies have provided empirical evidence for the operation of both mechanisms in the evolution of aging (8). Studies have also explored their operation in diseases including Alzheimer's disease (18–20)**.
**The statistical theory of extreme values (21–24)**
**1. The statistical theory of extreme values is concerned with the distribution of the maximum, the minimum, or other extreme order statistics derived from a collection of random variables following an initial distribution**.
**2. The cumulative distribution function (c.d.f.) of a random variable *X*, denoted by *F*(*x*), is the probability of observing a value of *X* no greater than *x*. In symbols, *F*(*x*) *= P*[*X* ≤ *x*]. Most of the common c.d.f.'s used in biological modeling are differentiable and for those, the derivative *f*(*x*) = *dF*(*x*) / *dx* is called the probability density function (p.d.f.) of *x*. If *X*_1_,..., *X_*n*_* are *n* independent observations from an initial distribution with c.d.f. *F*(*x*), the probability that each is no greater than *x* is {*F*(*x*)}*^*n*^*; this is the same as the probability that the maximum *M_*n*_*= max{*X*_1_,..., *X_*n*_*} is no greater than *x*. Therefore, {*F*(*x*)}*^*n*^* is the distribution function of *M_*n*_***.
**3. By the same reasoning, the probability that an observation with c.d.f. *F*(*x*) exceeds *x* is given by 1 – *F*(*x*); this is called the survival function and is denoted by *S*(*x*) = 1 – *F*(*x*) = *P*[*X* > *x*]. The probability that *n* independent observations *X*_1_,..., *X_*n*_* from the initial distribution all exceed *x* is {1 – *F*(*x*)}*^*n*^* = {*S*(*x*)}*^*n*^;* this is the same as the probability that the minimum *m_*n*_*= min{*X*_1_,..., *X_*n*_*} exceeds *x*. Therefore, {*S*(*x*)}*^*n*^* is the survival function of the minimum *m_*n*_***.
**4. If the independent observations come from *n* not necessarily identical initial distributions, say *F*_1_(*x*),..., *F_*n*_*(*x*), the c.d.f.'s of the maximum and minimum are given, respectively, by *F*_1_(*x*)···*F_*n*_*(*x*) and 1 – {*S*_1_(*x*)}···{*S_*n*_*(*x*)}**.
**5. The above expressions pertain to the *exact* statistical theory of extreme values and show that the distributions of the maximum or minimum depend both on the initial distribution and on *n*. The *asymptotic* statistical theory of extreme values covers results about the *limiting* distribution of an extreme order statistic as *n* tends to infinity under some centering and scaling of the extreme order statistic**.
**6. Centering and scaling is required because for any fixed value of *x*, lim *_*n* → ∞_ F ^*n*^*(*x*) = 0 or 1 if, respectively, *F*(*x*) <1 or *F*(*x*) = 1, i.e., the limiting distribution of the maximum is degenerate (similarly for the minimum). Thus, a non-degenerate limiting distribution must be found as the distribution of some sequence of transformed values of the maximum (or minimum) that depend on *n* but not on *x*.**
**7. For a given *F*(*x*), if such a limiting distribution of the maximum (or minimum) exists and we denote it *G*(*x*), we say that *F* is in the maximum (or minimum) *domain of attraction* of *G*. For example, if *X*_1_,..., *X_*n*_* are independent observations with an exponential distribution with c.d.f. *F*(*x*) = 1 – exp(–*x*), then *n* times the minimum *m_*n*_* has a limiting distribution which is also exponential, so *F* is in the minimum domain of attraction of itself (though this is usually not the case for other distributions)**.

Second, component causes are considered to occur as random events following time-to-event distributions. Although Rothman (3) conceived of a sufficient cause in a deterministic way, other authors have recognized that the SCC model is not inherently deterministic (26–28). The time to event of the component cause in the EBMC is the age at which the component cause expresses its necessary causal role. For genetic factors, the time-to-event distribution of the component cause is generated by the inherent variability of biological phenomena, and genetic effects are “age-specific” in the sense that their expression is more common at given ages than others, in such a way that they have unimodal time-to-event distributions. For environmental factors, the time-to-event distribution of the component cause is generated by randomness in the timing and amount of exposure, such that the age of expression occurs when the cumulative exposure reaches a (possibly random) threshold.

Since a sufficient cause is only complete once all component causes express themselves, the time-to-event distribution of the sufficient cause is the distribution of the *maximum* age of expression of its component causes. Moreover, because there are arguably many sufficient causes for an aging-related disease or death due to intrinsic causes, the time-to-event distribution for disease incidence or intrinsic mortality in the population is the distribution of the *minimum* time to event of the sufficient causes. From the exact statistical theory of extreme values, the time-to-event c.d.f. of the sufficient cause is the product of the time-to-event c.d.f.'s of the component causes, assuming that the distributions of the component causes are independent (Table 1). Then, the survival function for disease incidence or intrinsic mortality in the population, assuming that the distributions of the sufficient causes are independent, is the product of the survival functions of the sufficient causes. We note that the assumption of independence is not reasonable for sufficient causes because they may share component causes, but more general results have been obtained for a large number of sufficient causes under reasonable assumptions, as we will discuss below when addressing the Gompertz mortality model.

**Figure 1:**
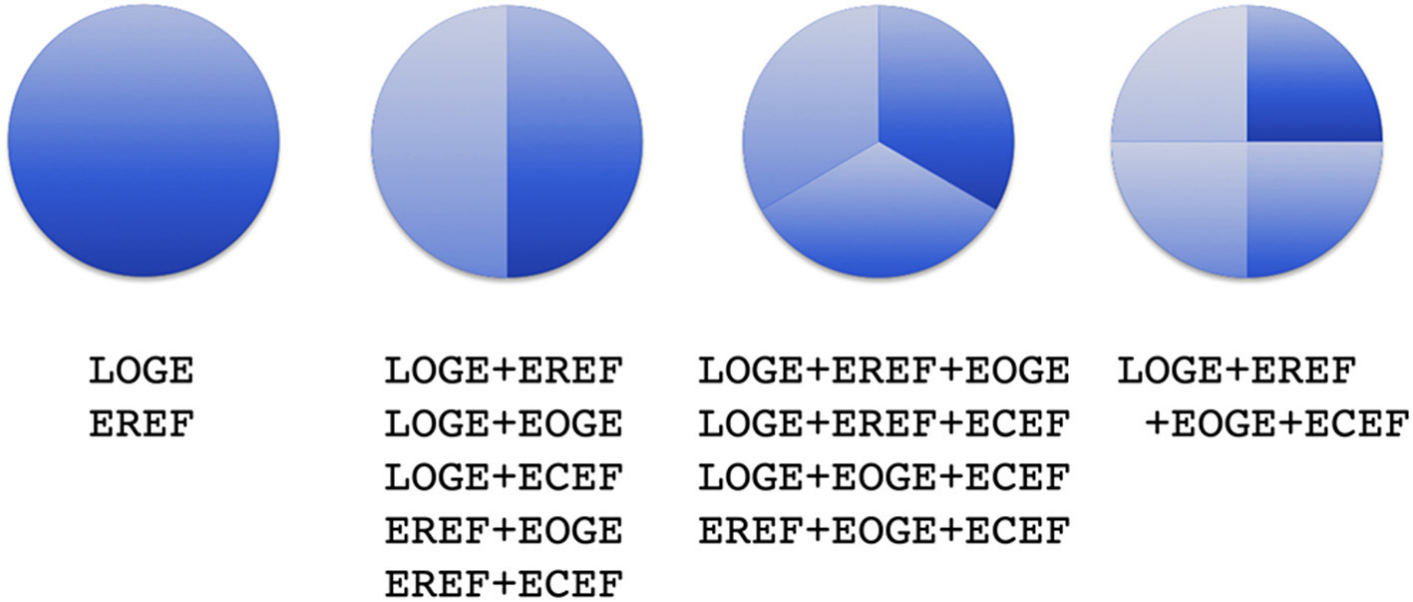
Schematic representation of 12 types of sufficient causes for aging-related diseases and intrinsic mortality using “causal pies.” Usually in this kind of representation of the sufficient and component causes model of causation, a pie represents a sufficient cause and the slices in each pie represent specific component causes, which can be a genetic or an environmental factor. A given genetic or environmental component cause can be shared by two or more sufficient causes. Here, under the evolution-based model of causation, the slices represent categories of component causes and the presence of a category of component cause in a sufficient cause means that one or more genetic effects or environmental factors of that category are part of the sufficient cause. Moreover, genetic and environmental component causes are considered to occur as random events following time-to-event distributions. Aging-related diseases are taken to involve at least hundreds of sufficient causes, as currently supported for cancer (30) and neurodegenerative disease (31); a monogenic form of an aging-related disease corresponds to a sufficient cause containing only one LOGE. ECEF, evolutionarily conserved environmental factor(s); EOGE, early-onset genetic effect(s); EREF, evolutionarily recent environmental factor(s); LOGE, late-onset genetic effect(s) [reproduced from Levy and Levin (1)].

The four categories of component causes can combine into 15 types of sufficient causes involving one to four categories. The EBMC is concerned with the evolutionarily-defined aging phase of life (the other phases being development and late life) (29), which starts at the earliest age of reproduction in the population. Thus, we exclude from consideration sufficient causes containing only EOGE or ECEF, or both EOGE and ECEF, because either by definition or by assumption they express themselves before the earliest age of reproduction in the population. If EOGE and/or ECEF participate in a sufficient cause also containing LOGE and/or EREF, the expression of the sufficient cause occurs after the earliest age of reproduction as the age of expression of the sufficient cause is given by the *maximum* age of expression of its component causes. The remaining 12 types of sufficient causes are schematically represented in Figure 1. EREF component causes may express themselves before or after the earliest age of reproduction in the population, but the sufficient causes in which they participate are only taken into account in the EBMC when these sufficient causes express themselves after the earliest age of reproduction.

Importantly, sufficient causes containing LOGE (without EREF) components have been subjected to natural selection over evolutionary history as a consequence of LOGE being heritable factors. This is not the case for sufficient causes containing EREF components, even if these sufficient causes may also contain heritable genetic components, because the EREF component causes necessary for their phenotypic expression only came into play relatively recently on an evolutionary scale, such that natural selection has not acted on them long enough. We thus derive a classification of sufficient causes according to whether or not their time-to-event distributions are “molded” by the declining force of natural selection: sufficient causes containing LOGE (without EREF) and those containing EREF (with or without LOGE), respectively, have time-to-event distributions molded and not molded by the declining force of natural selection. Therefore, the distinguishing characteristic is the absence or presence of EREF. As we show below, we obtain for each of the two groups different time-to-event distributions for disease incidence or intrinsic mortality asymptotically (i.e., for a large number of sufficient causes), based on results from the asymptotic statistical theory of extreme values and further evolutionary reasoning.

## Explanatory Power for Aging-Related Diseases

According to the principle of scientific inference known as “inference to the best explanation,” explanatory power derives from the degree to which a hypothesis or theory possesses explanatory virtues, including depth, unification or breadth, simplicity, and coherence with background scientific knowledge (32). The EBMC was not developed with a view to providing explanations for observations about aging-related diseases, but rather within the broad context of quantifying and elucidating the meaning of aging-relatedness (1). However, it does provide satisfactory explanations for several observations. Some of these observations have had explanations offered previously, but unlike the ones that we describe below, they were *ad hoc* and not connected by a unifying framework.

### Observation 1: Common Genetic Risk Variants Have Low Penetrance While Rare Variants Have High Penetrance

The etiology of complex diseases remains largely unknown despite the use of genomewide association studies, mostly under the premise of the common disease-common variant hypothesis, according to which common diseases are caused by common genetic variants or polymorphisms that individually have little effect (33, 34). Common variants initially identified by genomewide association studies were responsible for only a small fraction of the genetic contribution supposed to exist for complex diseases (35), and there is now ample evidence that both common and rare variants play a role in their causation (36, 37). The EBMC provides a straightforward way to see why low-penetrance genetic risk variants are common and high-penetrance variants are rare (or, rather, how genetic variants have become common/rare over evolutionary history as a result of their low/high penetrance). Under the EBMC, the penetrance of a genetic variant, or the probability of disease given the expression of the variant, depends on the expression of the other genetic and environmental component causes in a sufficient cause. Since phenotypic expression is necessary for the action of natural selection, low-penetrance variants have become common because, in the absence of expression of their causal partners, the action of natural selection does not occur (as if the causal partners “shielded” them from the action of natural selection, in a similar way that recessive genes are not subjected to natural selection in non-affected carriers). On the other hand, high-penetrance variants, or at the extreme fully penetrant monogenic causes of disease, are rare because they need fewer or no causal partners to produce phenotypic expression, such that they have been more consistently subjected to natural selection over evolutionary history.

### Observation 2: There Are Monogenic Forms of Aging-Related Diseases With Earlier Age of Onset Than the Sporadic Forms

As exemplified by Alzheimer's disease, Parkinson's disease, and some types of cancer, there are rare early-onset forms of aging-related diseases caused by fully penetrant autosomal dominant or recessive mutations, while the more common late-onset forms are associated with common polymorphisms (30, 38). This observation has motivated the view of a “genetic dichotomy” according to which earlier-onset monogenic forms should be considered separately from sporadic forms (39, 40). Contrary to that, in the EBMC fully penetrant mutations are represented by sufficient causes containing a single LOGE component cause, along a continuum with sufficient causes involving a few or several genetic and environmental component causes (Figure 1). In contrasting Mendelian and complex diseases, Petronis (41) asked: “If gene mutations underlie complex diseases, why is the age of onset delayed by a number of decades, whereas mutations in simple diseases express relatively early?” He and others offered an explanation based on the age-dependency of epigenetic changes (41, 42). In principle, this explanation could also be applied within aging-related diseases to account for the earlier onset of monogenic forms. However, the EBMC offers a simpler, more parsimonious explanation. Since the time to event (age of onset) for each sufficient cause is given by the *maximum* time to event of the component causes, it follows that it tends to be smaller (earlier age of onset) for sufficient causes containing only one component cause compared to sufficient causes with several component causes.

### Observation 3: Age Is a Risk Factor for Aging-Related Diseases

The observation that age is a risk factor for a given disease is now commonplace, so much so that Kirkwood (43) stated that “Age is by far the biggest risk factor for a wide range of clinical conditions that are prevalent today.” Yet once we characterize a condition as an aging-related disease, this statement becomes a truism. Moreover, there is no general agreement as to what “age as a risk factor” means. Costa and McCrae (44) noted: “on a little reflection, it becomes clear that chronological age is not in itself an explanatory variable, but rather a kind of index, backdrop, or dummy variable which is used to stand for the process or processes which causally underlie the universal, progressive and deleterious changes which we call aging.” However, Peto and Doll (45), in an article subtitled “Old age is associated with disease, but does not cause it,” questioned the usefulness of the very notion of “aging process” in this context: “What the major diseases of adult life have shared for tens of millions of years is a common set of evolutionary pressures tending to relegate them to old age, but such relegation is likely to involve many different mechanisms.” Similarly, after noting that “The aging process is now the major risk factor for disease and death after around age 28 in the developed countries,” Harman (46) stated that “The importance of the aging process to our health and wellbeing is obscured by the protean nature of its contributions to non-specific change and to disease pathogenesis.” In the EBMC, the association of older age with higher disease risk is a corollary of the time-to-event distributions of the sufficient causes, i.e., the “effect” of age on the development of disease is a *property* of the time-to-event distributions of the sufficient causes. Thus, the observed association of age with disease is a consequence of the cumulative expression of component causes over time, rather than age being a component cause itself.

### Observation 4: The Most Frequent Aging-Related Diseases Tend to Have Later Age of Onset Than the Others

As the populations of advanced countries have aged, it has become evident that the more frequent a given aging-related disease is, the later its peak age-specific incidence rate tends to be. For example, among malignant neoplasms, the four most frequent types (excluding non-melanoma skin cancer) are lung cancer, colorectal cancer, breast cancer and prostate cancer (47, 48), all with peak incidence after age 70 years (49–53). Among neurodegenerative diseases, the three most frequent ones, amyotrophic lateral sclerosis, Parkinson's disease, and Alzheimer's disease in increasing order of frequency, have peak incidence at ages ≥60, ≥70, and ≥80 years, respectively (54). Under the EBMC, the relation between more frequent occurrence and later age of onset is explained by the fact that a disease with a set of sufficient causes with overall later onset than another has been subjected to a weaker force of natural selection, resulting in a higher frequency in the population. This explanation does not require genes specifically controlling or modulating the age of onset of aging-related diseases, as have been investigated for Alzheimer's and Parkinson's diseases (55) and amyotrophic lateral sclerosis (56). Genetic component causes influence both the risk and the age of onset of disease simply because they are random events with time-to-event distributions. Related to that, the time-to-event distribution of genetic component causes also accounts for the notion of age-specificity of genetic effects (Table 1). Wensink (57) discussed logical problems in a process proposed to underlie age-specificity that involved an independent somatic change triggering the expression of deleterious genes at late ages. In the EBMC, age-specificity is explained without resort to extrinsic factors, resulting from genetic effects having unimodal time-to-event probability density functions (p.d.f.'s).

### Observation 5: Aging-Related Diseases Associated With the Modern Western Lifestyle Have Emerged or Become More Frequent in the Twentieth Century

The concept of “diseases of civilization” is based on the idea that there is a mismatch between the human genetic makeup, which was conditioned by past environments, and modern life, in which lifestyle factors (e.g., nutrition and sedentarism) and exposure to noxious substances represent a dramatic departure from those past environments, leading to the emergence of diseases associated with the modern Western lifestyle (58). Omran (59) already hinted at that in his theory of epidemiologic transition, by noting that in “the age of degenerative and man-made diseases,” there is “a tendency to overnutrition including consumption of rich and high-fat foods which may increase the risk of heart and metabolic diseases.” Ischemic heart disease is an important example of disease of civilization, as evidenced by the hunter-gatherer indigenous Tsimani population of Bolivia having the lowest reported levels of coronary artery disease of any population recorded to date (60). In the EBMC framework, the effect of the mismatch between the human genetic makeup and modern life corresponds to the contribution of sufficient causes containing EREF to disease incidence. The implication that diseases of civilization can be prevented through changes in lifestyle and minimization of exposures (i.e., the reduction in the contribution of sufficient causes containing EREF) unifies two lines of thought: the notion of primary prevention based on evolutionary principles, or the so-called “evolutionary health promotion” (61), and the epidemiological approach described by Rose (62), within the “population strategy” of disease prevention, as “the restoration of biological normality by the removal of an abnormal exposure (e.g., stopping smoking, controlling air pollution, moderating some of our recently-acquired dietary deviations).”

## Explanation of the Gompertz Mortality Model

In 1825, the British mathematician and actuary Benjamin Gompertz proposed a relation between age-specific mortality rates and age in which death rates increased geometrically as age increased arithmetically. This exponential relation has become known as the Gompertz model or “law of mortality” (63, 64). While Gompertz's proposal was based on empirical human mortality observations, he speculated about an underlying physiological explanation, which he further elaborated in a paper presented to the International Statistical Congress in 1860 and reprinted posthumously (65, 66). The Gompertz model has become widely used in demography and gerontology, but no satisfactory explanation for it emerged at least through the end of the twentieth century, as reviewed by Olshansky and Carnes (67). This is reflected in Williams' (68) 1999 statement, “Despite its many decades of usage, this model is based entirely on intuition and data-fitting, rather than inference from biological principles.” Forty years earlier, Beard (69) had posited that “A satisfying basis for a law of mortality would be a formula that, starting from some fundamental concepts about the biological aging process, led to a distribution of deaths by age which was comparable with observational data.” In the early 1960's, two prominent theories about the biological basis of the Gompertz model drew inspiration from physicochemical processes and reasoned at the level of homeostatic systems (70, 71). More recently, Gavrilov and Gavrilova's (72, 73) reliability theory of aging and longevity relied on analogies with mechanical devices. Others similarly drew on reliability theory while adding a biological layer. For example, Abernethy (74, 75) developed a theoretical account at the level of biological components identified with “minimal subsets of cells such that every subset is demonstrably vital for survival” and Richmond and Roehner (76) took as a first step “to decompose any organism into its vital organs.” Beard's phrase, *starting from some fundamental concepts about the biological aging process*, indicates what has been lacking from these and other explanations of the Gompertz model.

It is convenient to characterize survival distributions in terms of the *hazard function, h*(*x*), which is the instantaneous risk of death at age *x* given survival up to age *x*. For distributions with p.d.f. *f* (*x*) and survival function *S*(*x*), the hazard function is simply *h*(*x*) = *f* (*x*)/*S*(*x*). The Gompertz model is given by *h*_*G*_*(x)* = λ*e*^θ*x*^, where *h*_*G*_*(x)* is the Gompertz hazard function, λ > 0 is a parameter denoting the initial mortality rate, and θ is an exponential rate parameter, also called the rate of aging or Gompertz parameter (1). A need for two restrictions in the application of the Gompertz model has been recognized based on empirical observations. First, the Gompertz model is suitable for mortality within a range of ages rather than over the entire human lifespan. At one extreme of life, mortality rates from birth through infancy decrease down to a minimum in the beginning of the second decade, which has been seen as a justification for estimating the initial mortality rate (parameter λ) at the age of puberty or sexual maturity (77, 78). At the other extreme, a deceleration of mortality rates or “late-life mortality plateau,” meaning a less-than-exponential increase in mortality rates, has been observed after about age 90 (67, 78, 79). Second, the Gompertz model applies to intrinsic mortality, i.e., excluding deaths due to accident, homicide, suicide, starvation, and infectious disease. The partitioning of mortality into extrinsic and intrinsic (80) was anticipated by Gompertz (63) (“It is possible that death may be the consequence of two generally co-existing causes; the one, chance, without previous disposition to death or deterioration; the other, a deterioration, or an increased inability to withstand destruction”) and derives from the work of British actuary William Makeham (81, 82), who proposed to incorporate an age-independent additive parameter to the Gompertz hazard function that closely accounts for the contribution of extrinsic causes to mortality (78, 83).

We provide an account of the Gompertz model at the fundamental level of genetic causes and involving evolutionary biology, the discipline par excellence underlying our understanding of how biological processes have come to be what they are (84). To that end, we consider the application of the EBMC to intrinsic mortality, and assume that there is a large enough number of sufficient causes of death due to intrinsic causes in the population such that the asymptotic statistical theory of extreme values provides accurate approximations; we have verified that the approximations are already reasonably accurate for 100 sufficient causes (1). An immediate benefit of an explanation of the Gompertz model under the EBMC is that, by relying on the evolutionary theory of aging, the restrictions above naturally arise from theoretical considerations. Based on the evolutionary theory of aging (Table 1), the Gompertz model is accounted here not merely in relation to the force of natural selection *per se*, but in connection with its decline with increasing age. Since the force of natural selection starts to decline at the earliest age of reproduction in the population and reaches zero at the end of reproduction (or rather at the end of the effective transfers of food and care) (4, 5), the Gompertz model would pertain to the corresponding in-between range of ages at death (29). Moreover, since the declining force of natural selection acts on age-specific genetic effects, such account excludes extrinsic causes of death. Under the EBMC, an additional restriction is imposed in that we must discount the contribution of sufficient causes of death containing environmental factors that are relatively recent on an evolutionary scale, because they cannot be regarded as constitutive of the environment in which evolution developed. Therefore, we restrict our account to sufficient causes containing LOGE (without EREF), corresponding to the group of sufficient causes whose time-to-event distributions have been molded by the declining force of natural selection.

Based on this, we can address two questions. The first is: Given that the intrinsic mortality distribution in the population is the distribution of the minimum time to event of the sufficient causes, is there a condition in which the distribution of the minimum of sufficient causes containing LOGE (without EREF) approaches the Gompertz distribution as the number of such causes becomes large? Some classical results in the statistical theory of extreme values show that the Gompertz distribution is one of only two possible asymptotic distributions of the minimum of independent and identically distributed time-to-event random variables (21, 85). These results include necessary and sufficient conditions on the distribution function of those random variables to be in the minimum domain of attraction of the Gumbel distribution (22, 86) (which becomes the Gompertz distribution when restricted to the positive half line), and the simpler and widely applicable sufficient condition obtained by von Mises (87). We showed that the von Mises condition for the minimum implies that the initial c.d.f. must have all derivatives equal to zero when evaluated at the lower terminus of the distribution [Corollary to Theorem 2 in reference (1)], a local condition we refer to below as “flat” at the lower terminus. We take the lower terminus to be the earliest age of reproduction in the population. Under reasonable additional conditions, we proved the converse, i.e., that a flat initial c.d.f. implies a limiting Gompertz distribution for the minimum [Theorem 6 in reference (1)]. Although the time-to-event random variables under our framework are neither independent (because sufficient causes may share component causes) nor identically distributed (because their distributions are the distributions of the maximum time to event of different sets of component causes), we additionally showed that we can relax those requirements under reasonable assumptions [Theorems 8 and 9 in reference (1)]. Thus, these results support the conclusion that if the initial distributions of sufficient causes containing LOGE (without EREF) belong to a family of flat functions at the earliest age of reproduction, the asymptotic distribution of the minimum is the Gompertz distribution.

The second question is: Is this condition met by the time-to-event distributions of sufficient causes containing LOGE (without EREF), in connection with them being molded by the declining force of natural selection? We argue based on evolutionary reasoning by starting with the notion that the probability of fixation of a deleterious mutation, hence its ultimate frequency in the population under mutation-selection balance, is inversely related to its age of expression (16). This is to say that, for example, a deleterious mutation that expresses its effect around age 60 years is subjected to weaker negative selection and is present at higher frequency in the population than a mutation that expresses its effect around age 40 years. Since phenotypic expression for the action of natural selection only occurs under the EBMC framework once a sufficient cause is complete, we apply this reasoning at the level of the sufficient causes containing LOGE (without EREF). Among these, sufficient causes with distributions shifted closer and closer to the earliest age of reproduction in the population would be present at successively lower frequencies, such that sufficient causes with time-to-event distributions shifted heavily toward the earliest age of reproduction, or presenting a relatively heavy lower tail, would tend to be eliminated through the action of natural selection over evolutionary history.

This is admittedly not enough to require time-to-event distributions of the sufficient causes that are flat at the earliest age of reproduction. However, we further consider the extreme dependence of human offspring on parental care for a relatively long period of time. For human ancestors, it is reasonable to suppose that if a parent died soon after childbirth, the prospects of survival of the offspring was greatly reduced (17, 88). Indeed, according to Williams (17), “In many primitive human societies the death of teen-age parents must have greatly reduced the survival prospects of any children they might have produced. The care of dependent offspring is as important to human reproduction as the production of gametes.” Under these conditions, the force of natural selection acting on a mutation that reduces survival during the early reproductive years is intensely sustained at or very near the maximum level. Then, one can argue that the strong negative selection on these genetic factors would effectively preclude all but sufficient causes with flat distributions at the earliest age of reproduction in the population. Williams (17) alluded to that by adding: “So the rate of decline in reproductive probability [a function he defined as depending on both survival and fecundity] in early adulthood must be very slight, and this factor should result in a very low rate of senescence during the first decade of man's reproductive life.” This evolutionary reasoning draws to a close the explanation of the Gompertz mortality model under the EBMC.

We further present here complementary results relevant to life expectancy in present human populations. The main drivers of the substantial increase in life expectancy in the twentieth century are related to the two restrictions above in the application of the Gompertz model. Improvements in sanitation and standards of living as well as public health immunization campaigns led to a decrease in infantile and childhood mortality and a decrease of mortality due to infectious disease over all ages, i.e., mortality involving ages and/or causes of death outside the applicability of the Gompertz model. The potential for further increases in life expectancy can be understood in relation to the additional restriction under the EBMC. On one hand, the explanation of the Gompertz model on the basis of sufficient causes containing LOGE (without EREF) implies that the Gompertz distribution (with a given rate of aging or Gompertz parameter θ) represents a benchmark set by the evolutionary process, in the sense that the schedule of intrinsic mortality is related to the declining force of natural selection and it is somehow “calibrated” to elements of human reproductive biology (e.g., the ages when reproduction starts and ends) as well as intergenerational transfers (5, 89, 90). This is consistent with recent evidence supporting the invariant rate of aging hypothesis, according to which the rate of aging is relatively constant within humans and other species (91). On the other hand, such an account implies that sufficient causes of death containing EREF underlie *departures* from the Gompertz distribution (92–94), or as put by Major Greenwood long ago, “the blurring effect of the ‘environmental' factors which it cannot be supposed adequately to express” (95).

The other asymptotic distribution of the minimum of independent and identically distributed time-to-event random variables is the Weibull distribution, which was developed by the Swedish engineer Waloddi Weibull for modeling the strength of materials (96, 97). The Weibull hazard function describes a power relation between age-specific mortality (or failure) rates and age, given by *h*_*w*_(*x*) = αγ*x*^γ*-1*^, where α > 0 and γ > 0 (α^−1^ is a scale parameter and γ is a shape parameter). The necessary and sufficient condition for an initial distribution to be in the minimum domain of attraction of the Weibull distribution implies that the initial distribution is “regularly varying” at the lower terminus [defined in reference (1), p. 24]. Regularly varying distributions comprise a fairly wide class among “well-behaved” distributions. Karamata (98) showed that if the limit as *t* goes to 0 of the ratio *F(xt)/F(t)* exists for all *x* > *0*, then the hazard function of the asymptotic distribution of the minimum must be proportional to *x*^ρ^ for some real number ρ. The case ρ < *0* does not apply to non-decreasing functions such as c.d.f.'s. A value of ρ that is either zero or infinity (the latter corresponding to *F* flat at 0) are very special cases; absent some active process like molding by natural selection, such cases should not occur. Thus, a non-zero finite value of ρ should be the norm and these are precisely the cases where *F* varies regularly at 0 (or more generally at the lower terminus). Plausibly, then, sufficient causes not molded by the declining force of natural selection have a regularly-varying behavior at the lower terminus. Additional results allowing relaxation of the requirements of independence and identical distribution [Theorems 4 and 10 in reference (1)] support the conclusion that, if the initial distributions of sufficient causes containing EREF belong to a family of regularly varying functions at the lower terminus, the asymptotic distribution of the minimum is the Weibull distribution.

Table 2 summarizes the results presented in this section; these results are also valid for the incidence of specific aging-related diseases, assuming only that the number of sufficient causes for a given disease is sufficiently large for the asymptotic theory to apply as a good approximation, which is currently supported for cancer (30) and neurodegenerative disease (31) (as noted previously, we have verified that the approximations are already reasonably accurate for 100 sufficient causes). We further used these results to accurately model intrinsic mortality as a mixture of the Gompertz and Weibull survival distributions by analyzing a mortality dataset obtained over a 43-year follow-up period (1963–2006) (1). In 1932, in a paper titled “On some experiments in the graduation of mortality statistics” published in the *Journal of the Institute of Actuaries* (99), Wilfred Perks asserted with respect to mortality data: “Most of us retain, consciously or unconsciously, a feeling that, underlying all the roughnesses in our data referable to errors of observation and an ever-changing environment, there may be an inherent mathematical system of law and order, which if it could but be discovered would give such insight into the meaning of the unadjusted figures that a considerable advance would be made in the practical application of our science.” Under the EBMC, the “inherent mathematical system” is given by the Gompertz distribution and the “unadjusted figures” additionally include deaths due to EREF, reflected in the contribution of the Weibull distribution to mortality statistics; we will discuss a “practical application” from that vantage point after we consider the limitations of the EBMC.

**Table 2:** Time-to-event distribution for disease incidence or intrinsic mortality according to whether or not the time-to-event distributions of the sufficient causes are molded by the declining force of natural selection^a^.

**Classification of sufficient causes**	**Defining category of component causes**	**Time-to-event distributions of the sufficient causes**	**Evolutionary reasoning**	**Asymptotic time-to-event distribution^b^**
1. Time-to-event distributions molded by the declining force of natural selection	LOGE (without EREF)	Family of flat functions at the lower endpoint of the distribution	Intensely sustained force of natural selection in the initial years after the earliest age of reproduction (due to the extreme dependence of human offspring on parental care for a relatively long period of time) leads to flat behavior at the lower endpoint	Gompertz distribution
2. Time-to-event distributions not molded by the declining force of natural selection	EREF (with or without LOGE)	Family of regularly-varying functions at the lower endpoint of the distribution	Absence of molding by force of natural selection leads to regularly-varying behavior at the lower endpoint	Weibull distribution

a *LOGE, late-onset genetic effect(s); EREF, evolutionarily recent environmental factor(s).*

b *For large numbers of sufficient causes*.

## Limitations of the EBMC

As all theoretical models, the EBMC is a useful simplification of reality that has potential limitations. Some limitations are just restrictions in the applicability of the model. For example, the distributional results described in the previous section are asymptotic, which is to say that they apply to diseases with a large number of sufficient causes. This is not the case for Mendelian diseases, including those with onset in adult life like Huntington's disease. We established this limitation at the outset by defining aging-related diseases as diseases of complex etiology and stating that the EBMC is only applicable to aging-related diseases and intrinsic mortality. Aside from these limitations, some more serious limitations may emerge through observations that apparently contradict the basic premises of the model. Others may emerge when the model is confronted with related theoretical developments that are themselves well-supported by observations. So far, to the best of our knowledge, the EBMC has been able to accommodate both kinds of challenges.

For instance, in the previous section we referred to evidence supporting the invariant rate of aging hypothesis, according to which the rate of aging is relatively constant within humans and other species, as being consistent with the explanation of the Gompertz model under the EBMC. In the cited study, Colchero et al. (91) presented the results of an analysis of 30 mortality datasets pertaining to six genera of non-human primates along with the results for nine human mortality datasets. By fitting a five-parameter general mortality function to the data, the analysis showed that within each primate genus and across human populations the rate of aging parameter (corresponding to Gompertz parameter θ) varied very little and orders of magnitude less than the other mortality parameters. On the other hand, another recent study showed that total daily energy expenditure, a variable that may be considered a physiological measure of aging, remains stable between 20 and 60 years and then declines in older adults (100), suggesting a non-constant rate of aging over the life course. This could be regarded as an observation against the explanation of the Gompertz model under the EBMC, because of the implication of a fixed Gompertz parameter governing the exponential growth in mortality rate as a function of age, but it is unclear how a varying energy expenditure over the life course of an individual would manifest in mortality at the population level.

To show how the EBMC can accommodate related theoretical developments, we consider the fields of life course epidemiology and epigenetics. A life course approach to chronic disease epidemiology is defined as “the study of long-term effects on chronic disease risk of physical and social exposures during gestation, childhood, adolescence, young adulthood and later adult life” (101). Despite the interest devoted to early life exposures, the life course approach does not undermine the importance of midlife lifestyle or other risk factors to disease. Moreover, the contribution of early life exposures to chronic aging-related diseases can be taken into account in the EBMC, even if the model is only concerned with disease onset after the earliest age of reproduction in the population. According to Kuh et al. (102), “The purpose of life course epidemiology is to build and test theoretical models that postulate pathways linking exposures across the life course to later life health outcomes.” Such pathways lay out the temporal ordering of exposures, their inter-relationships, and connections with the outcome measure (101, 102). The life course approach “explicitly recognizes the importance of time and timing in understanding causal links between exposures and outcomes” (103). In the EBMC, the relationship among exposures and the importance of time are implicitly recognized through the consideration of sufficient causes as sets of component causes with time-to-event distributions.

There is increasing evidence that cellular epigenetic mechanisms (e.g., DNA methylation and histone modifications) are relevant to disease causation (104, 105). The term *epigenetics* is most often used in the sense of mitotically and meiotically heritable changes in gene expression that are not coded in the DNA sequence (106). While the transmission of induced changes in epigenetic states is crucial for normal development, Holliday (107) considered that epigenetic defects might also contribute to the risk of disease, and coined the term *epimutation* to designate “the heritable changes based on DNA modification,” as distinguished from mutations that are changes in DNA sequence. Epigenetic modifications provide a link between the environment and alterations in gene expression that may lead to disease phenotypes (108). Epigenetic mechanisms respond to levels of dietary and metabolic precursors and cofactors for methylation and acetylation (109, 110), which can account for diet effecting epigenetic changes. The list of environmental factors that can result in epigenetic changes also includes drugs, xenobiotic chemicals, smoking, endocrine disruptors, heavy metal toxins, and low-dose radiation (108, 110–115). In the EBMC, epigenetic mechanisms represent an alternative route for the action of environmental component causes involving modification of patterns of gene expression, as opposed to a “direct” effect on disease causation. Such epigenetic mediation of environmental effects can involve mitotic inheritance of environmentally-induced epigenetic changes at any time within an individual's lifetime. In case environmental induction occurs early in life, even if it occurs in intrauterine or early postnatal life, the EBMC can take it into account as long as it has sustained effects on gene expression.

## Discussion: Implications for Healthy Aging

A premise of the influential compression of morbidity hypothesis was that the “rectangularization” of the survival curve for mortality would proceed in connection with the elimination of “premature deaths” (116). In our framework, deaths caused by sufficient causes containing EREF are premature in the sense that they occur earlier than they would under the Gompertz model, and the elimination of premature deaths involves primary prevention through changes in lifestyle and reduction of environmental exposures. We refer to this here as “health promotion.” While the increase in life expectancy in the twentieth century was due to a redistribution of deaths from the young to the old, health promotion involves delaying deaths within older ages, with a much smaller impact on life expectancy. However, this is not to say that the potential gains are negligible. As suggested by observed departures from the Gompertz model (92–94), the returns from preventing premature deaths can be substantial due to across-the-board effects of risk factor modification on several diseases (117–119). An analysis of the age at which the remaining life expectancy fell to 10 years in some countries provided additional evidence that delaying deaths within older ages can produce substantial gains; for Swedish and Japanese women, respectively, this age rose by about 8 and 12 years between 1950 and 2008 (120). Still, despite repeated assertions that life expectancy was approaching a ceiling having been proven wrong (121), the supposition that advanced countries may now be approaching a biologic limit to life expectancy has motivated a call for preferential investment in “postponed aging” (46, 122–124). Postponed aging requires a modification of the fundamental processes of aging or “manipulating ‘aging' genes through techniques developed in molecular biology” (125).

The promises of health promotion and postponed aging have similarly been recognized for the goal of promoting healthy aging. As stated by Brody (126) in 1985 and still valid today, we are “far short of reaching the potential of better health and quality of life in later years. Two vehicles offer promise: health promotion through the improvement of personal health practices throughout life, and major research efforts to understand and postpone the aging processes.” In order to examine these two scenarios under the EBMC, we define “healthy aging” as aging in the absence of aging-related diseases. Another premise of the compression of morbidity hypothesis was that there would be a postponement of the onset of chronic diseases through lifestyle changes, resulting in “rectangularization not only of the mortality curve but also of the morbidity curve” (116); yet compression of morbidity additionally depends on morbidity gains exceeding mortality gains, such that the area between the morbidity and mortality curves is “compressed” over time (127). Some studies have supported instead the occurrence of an *expansion* of morbidity in recent decades (125, 126, 128), but this is likely related to a predominance of secondary and tertiary preventions (controlling disease progression and averting fatal complications) over primary prevention in the populations under study (127). In longitudinal studies of chronic aging-related conditions (129), cardiovascular disease (130), and dementia (131, 132), the evidence in favor of the potential of compression of morbidity through primary prevention is compelling. Longitudinal studies have also demonstrated the occurrence of less years of disability at the end of life for subjects with a lower number of lifestyle risk factors including smoking, physical inactivity, and obesity (133, 134), and especially for those who engaged in vigorous aerobic exercise at middle and older ages (135, 136). Proponents of postponed aging argue that it might also decrease the duration of unhealthy years (46, 123, 124), but evidence for that remains to be produced (137).

**Figure 2:**
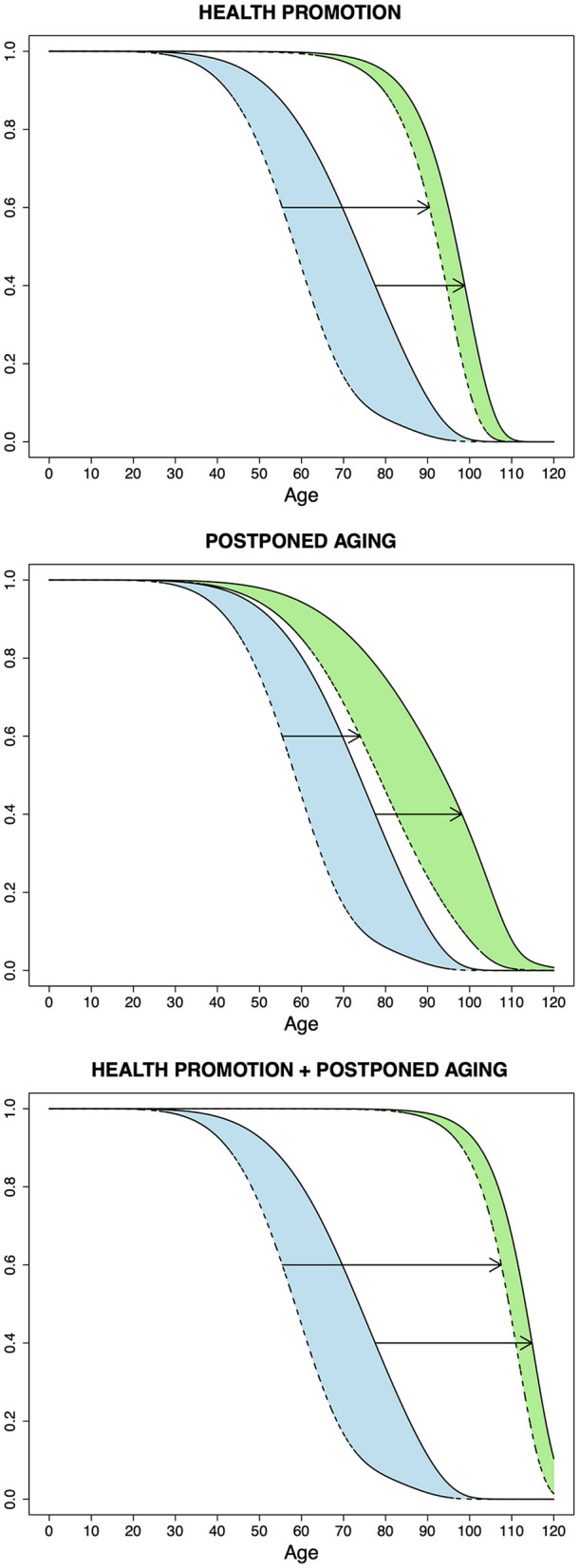
Prospects for healthy aging according to the scenarios of health promotion, postponed aging, and the two scenarios combined, using hypothetical survival functions for incidence of aging-related diseases (dashed lines) and for intrinsic mortality (continuous lines). The upper and lower arrows show the shift in the morbidity and mortality curves, respectively, under each scenario. The colored areas correspond to years lived with disease or unhealthy years; this is represented before (blue) and after (green) achieving the outcome in each of the scenarios. A reduction in these areas is consistent with the compression of morbidity hypothesis and occurs under health promotion and the two scenarios combined. The Gompertz distribution was used for the outer “rectangularized” morbidity and mortality curves in the top and bottom graphs; all other curves were drawn using a mixture of the Gompertz and Weibull distributions.

In Figure 2, we represent the prospects for healthy aging according to the scenarios of health promotion and postponed aging as well as the two scenarios combined, using hypothetical survival functions for incidence of aging-related diseases and mortality (morbidity and mortality curves, respectively). A curve is said here to “shift to the right” as a result of a higher proportion of disease or death occurring at older ages. Based on the previously presented results (Table 2), the Gompertz distribution was used for the “rectangularized” morbidity and mortality curves, presuming complete elimination of EREF, and all other curves were drawn using a mixture of the Gompertz and Weibull distributions. Under health promotion, both the morbidity and mortality curves shift to the right but the morbidity curve is shifted further. There is a decrease in unhealthy years at the same time that there is an increase in longevity (consistent with the compression of morbidity hypothesis), resulting in a substantial gain in healthy years. Under postponed aging, involving modification of the fundamental processes of aging, we represent the outcome in which the morbidity and mortality curves are shifted to about the same extent. In this case, there is a gain in healthy years but the duration of unhealthy years is not reduced. Under the two scenarios combined, both curves shift to the right with the morbidity curve shifting further, as in health promotion alone, but the rectangularized morbidity and mortality curves are displaced to a larger extent in relation to the modification of the aging process.

Overall, the implications for healthy aging under the EBMC framework underscore the value of health promotion. This is even more so because, although the two scenarios combined produce the most favorable outcome, evolutionary theory suggests that hundreds or thousands of genes and hundreds of biochemical pathways play a role in aging, indicating that postponed aging will remain a challenging and unfruitful strategy for many years to come. Different authors have expressed this view with high degrees of fatalism. According to Williams (17), the evolutionary theory implication of a large number of physiological processes involved in aging “banishes the ‘fountain of youth' to the limbo of scientific impossibilities where other human aspirations, like the perpetual motion machine and Laplace's ‘superman,' have already been placed by other theoretical considerations.” Wallace (138) noted that it is “futile to waste time searching for a single root cause of aging and its cure, an elixir vitae.” Rose (139) considered that the evolutionary perspective that aging results from a failure of adaptation involving a large number of genetic variants and biochemical pathways may lead one to “regard the slowing of human aging as an essentially intractable problem.”

## Conclusion

According to VanderWeele and Hernán (140), “Despite its seeming lack of utility in actual applications and data analysis, the sufficient-component cause model continues to be routinely taught in introductory epidemiology courses because it provides a useful framework in which to think about the actual causal mechanisms at work in bringing about a particular outcome.” Similarly, we have described the explanatory properties of the EBMC framework, but the EBMC may turn out to be useful for data-analytical applications as well, since it plays an essential role in the formulation of an index of aging-relatedness and its interpretation in terms of genetic and environmental contributions to disease incidence and mortality (1). Most importantly from a public health standpoint, the EBMC implies that primary prevention through changes in lifestyle and reduction of environmental exposures is paramount in promoting healthy aging. This is reinforced by declines in the incidence of aging-related diseases in a timeframe inconsistent with broad genetic changes in the population, as observed for ischemic heart disease starting in the 1960s, when education efforts were directed at smoking and diet (141), as well as for dementia and Alzheimer's disease more recently (142, 143).

Admittedly, lifestyle changes are difficult to implement both for the individual and at the societal level and we cannot go back to the pre-industrial environment of our ancestors. Still, the theoretical developments presented here support the continued implementation of public-health programs aimed at lifestyle changes and reduction of environmental exposures, even as the postponed aging scenario can be simultaneously pursued. After all, as bluntly stated by the father of the evolutionary theory of aging, Sir Peter Medawar, in an article titled “The future of life expectancy” (144), “No one entertains the ambition to populate the world with decrepit old dotards: what is hoped for is a readjustment to the tempo of aging such that a person of 90—of four score years and 10—has the same vigor and address to life as present-day 70-year-olds, and so proportionately at other ages.”

## Data Availability Statement

The original contributions presented in the study are included in the article/supplementary material, further inquiries can be directed to the corresponding author.

## Author Contributions

GL conceptualized and wrote the manuscript. BL contributed to the development of the ideas and revised the manuscript for intellectual content. All authors contributed to the article and approved the submitted version.

## Conflict of Interest

The authors declare that the research was conducted in the absence of any commercial or financial relationships that could be construed as a potential conflict of interest.

## Publisher's Note

All claims expressed in this article are solely those of the authors and do not necessarily represent those of their affiliated organizations, or those of the publisher, the editors and the reviewers. Any product that may be evaluated in this article, or claim that may be made by its manufacturer, is not guaranteed or endorsed by the publisher.
